# Defect and Doping Co-Engineered Non-Metal Nanocarbon ORR Electrocatalyst

**DOI:** 10.1007/s40820-020-00579-y

**Published:** 2021-02-06

**Authors:** Jian Zhang, Jingjing Zhang, Feng He, Yijun Chen, Jiawei Zhu, Deli Wang, Shichun Mu, Hui Ying Yang

**Affiliations:** 1 Key Laboratory of Material Chemistry for Energy Conversion and Storage, Ministry of Education, School of Chemistry and Chemical Engineering, Huazhong University of Science and Technology, Wuhan, 430074 People’s Republic of China; 2 Pillar of Engineering Product Development, Singapore University of Technology and Design, 8 Somapah Road, Singapore, 487372 Singapore; 3 State Key Laboratory of Advanced Technology for Materials Synthesis and Processing, Wuhan University of Technology, Wuhan, 430070 People’s Republic of China; 4 Foshan Xianhu Laboratory of the Advanced Energy Science and Technology Guangdong Laboratory, Xianhu Hydrogen Valley, Foshan, 528200 People’s Republic of China

**Keywords:** Defect, Doping, Electrocatalyst, Oxygen reduction reaction, Non-metal nanocarbon

## Abstract

Exploring low-cost and earth-abundant oxygen reduction reaction (ORR) electrocatalyst is essential for fuel cells and metal–air batteries. Among them, non-metal nanocarbon with multiple advantages of low cost, abundance, high conductivity, good durability, and competitive activity has attracted intense interest in recent years. The enhanced ORR activities of the nanocarbons are normally thought to originate from heteroatom (e.g., N, B, P, or S) doping or various induced defects. However, in practice, carbon-based materials usually contain both dopants and defects. In this regard, in terms of the co-engineering of heteroatom doping and defect inducing, we present an overview of recent advances in developing non-metal carbon-based electrocatalysts for the ORR. The characteristics, ORR performance, and the related mechanism of these functionalized nanocarbons by heteroatom doping, defect inducing, and in particular their synergistic promotion effect are emphatically analyzed and discussed. Finally, the current issues and perspectives in developing carbon-based electrocatalysts from both of heteroatom doping and defect engineering are proposed. This review will be beneficial for the rational design and manufacturing of highly efficient carbon-based materials for electrocatalysis.

## Highlights

- Recent advances of non-metal nanocarbon materials for electrocatalytic oxygen reduction reaction (ORR) are comprehensively summarized in terms of co-engineering of heteroatom doping and defect inducing.
- The characteristics, ORR performance, and the related mechanism of non-metal nanocarbon are emphatically analyzed and discussed.
- The current issues and perspectives in developing carbon-based electrocatalysts from both of heteroatom doping and defect engineering are pointed out and proposed.

## Graphical abstract

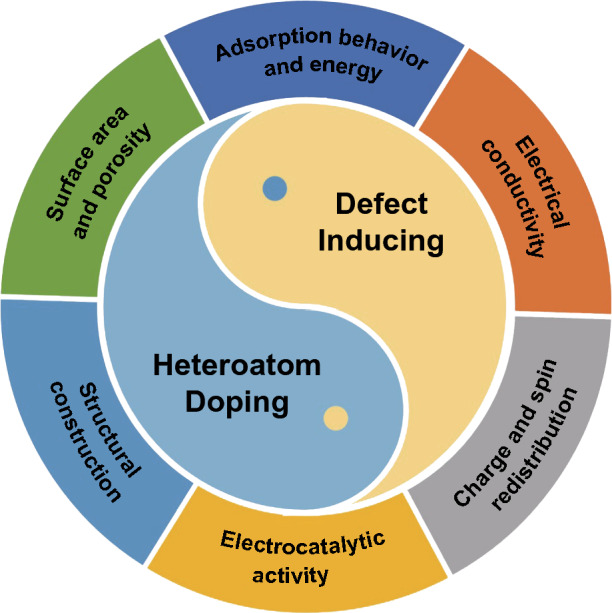

## Introduction

With the accelerated depletion of fossil fuels and the series of environmental issues, advanced energy conversion technologies have attracted considerable attention in academia and industry. Fuel cells and metal–air batteries are regarded as the next generation of clean power energy, because of their ability to directly convert the chemical energy of fuels to electricity directly [1–4]. However, the oxygen reduction reaction (ORR) in the cathode of fuel cells and metal–air batteries extremely suffers from sluggish kinetics, high overpotential, and poor stability, which requires catalysts to boost the reactivity [2, 3, 5–9]. The noble metal platinum (Pt) and its alloys have long been considered as the state-of-the-art catalyst for ORR; however, the drawbacks such as limited resources, prohibitive cost, susceptibility to fuel (e.g., methanol crossover and CO deactivation) and poor durability have greatly limited their large-scale commercial applications [4, 10–14]. Accordingly, exploring high efficiency, low cost of earth-abundant alternatives has aroused great interest in the past few decades.

**Fig. 1:**
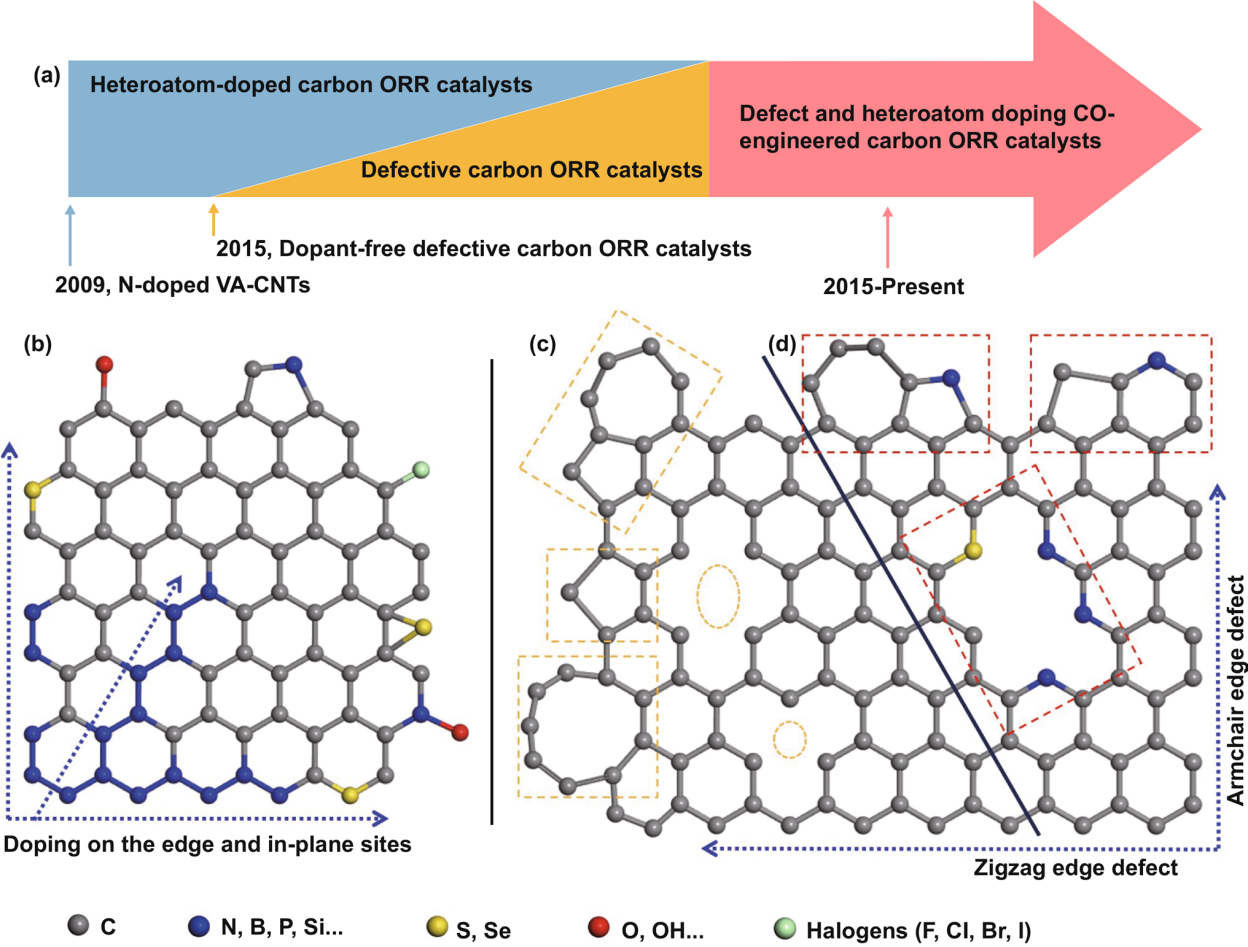
**a** Timeline showing the important developments of various functionalized non-metal carbon-based ORR electrocatalysts. Schematic illustration of the representative configurations: **b** heteroatom doping engineered carbon. **c** Defect-inducing engineered carbon. **d** Defect- and heteroatom co-engineered carbon

Among all the candidates, the functionalized carbon nanomaterials due to their earth abundance of resource, facile fabrication, high physicochemical stability, outstanding activity, fuel immunity, and environmental friendliness have been intensively studied and regarded as one of the most promising ORR catalysts [4, 12, 15–18]. Figure 1a approximately shows the timeline for the important developments of carbon-based metal-free ORR electrocatalysts. Pioneering work was reported by Dai’s group in 2009 on nitrogen-doped vertically aligned carbon nanotubes (N-doped VA-CNTs) for ORR [3]. After that, various heteroatom (N, B, S, O, P, F, etc.) single/dual/multiple doped non-metal nanocarbon ORR electrocatalysts have been investigated in succession. Owing to the incorporated heteroatoms with different electronegativity, the local charge and spin density of the carbon matrix have been significantly altered, which can facilitate the oxygen adsorption, decrease the reaction energy barrier, and subsequently break the O–O bond, enhancing the electrocatalytic ORR activity of such heteroatom-doped carbon nanomaterials [12, 15–24].

In the past few years, tremendous advanced heteroatom-doped carbon ORR nanomaterials have been made and showed excellent electrocatalytic ORR activity. Along with the deepening investigation of heteroatom doping effects, in 2015, the researchers found that even the non-doped defective carbon nanomaterials also possessed considerable ORR activity [2, 10, 22, 25–35], which could be comparable or even superior to heteroatom-doped carbon electrocatalysts. This is a great progress of understanding the mechanism of the carbon-based ORR catalyst. Based on the second law of thermodynamics [22, 36], carbon materials always have defects or disorder structure in the edge or surface. The absence of carbon atoms and/or the reconstructed lattice structures can break the electron–hole symmetry undoubtedly and possess higher charge and spin densities in comparison with ordinary carbon atoms [26, 29, 33, 37–44]. The defect engineering seems to have the same effect with the heteroatom doping. However, in most instances, the ORR activity of the non-doped defective carbon nanomaterials is inferior to that of doped carbons, despite a few exceptions [28, 40, 45–47]. When combined the defects with dopants, remarkably, the obtained catalysts usually reveal the highest ORR activity, indicating that the defects and dopants can contribute together or synergistically promote the electrocatalytic activity for nanocarbon.

Recently, although the work of heteroatom-doped [4, 12, 15–18, 22, 23, 48–50] or defect-induced nanocarbons for ORR [2, 5, 10, 25, 26, 47, 51–55] have been reviewed from the aspect of materials design, synthesis, and the related mechanism, a few reports are systematically and comprehensively concerned for the reviews on nanocarbon ORR electrocatalyst from co-engineering of heteroatom doping and defect inducing. In this regard, understanding the synergistic promotion effect of heteroatom doping and defect inducing, and their relationship between the electrocatalytic performance of various carbon-based materials is indispensable, which is significantly important to accelerate the development of the ideal carbon-based catalysts toward ORR.

Figure 1b–d reveals some representative configurations of heteroatom-doped, defect-induced, and defect-/heteroatom-co-engineered nanocarbons, respectively. Heteroatom doping is the substitution of carbon atoms with another non-carbon atom, such as B, N, P, S, F, O, and halogens. It promotes the electrocatalytic activity by altering the charge and spin redistribution among the around carbon atoms [12, 16, 17, 21, 22]. The defects in carbon nanomaterial include edges (typically zigzag and armchair edges), vacancies, pores, and various topological defects (non-hexacyclic-ring system like pentagon, heptagon, Stone–Wales defects, and so on). The introduction of defects could induce the structural distortion of carbon and break the integrity of *π* conjugation, leading to improved electrocatalytic activities [2, 5, 10, 25, 47, 51, 52, 54–56]. In practice, the carbon nanomaterials often contain both heteroatom dopants and defect sites. Particularly, when the preformed carbon nanomaterials undergo a post-heteroatom doping process with dopant-containing precursors, these heteroatoms are easier to locate at the edge/defect or substitute the edge carbon atoms instead of replacing the carbon at the perfect basal plane [2, 21, 25, 51, 57]. The optimal electrocatalytic active sites are hence tended to be formed at the combination/junction of heteroatom dopants and defects (Fig. 1d).

In this review, we start with a brief introduction of heteroatom doping for the development of carbon-based ORR electrocatalysts. Afterward, we present some typical examples of ORR from the aspect of constructing various defects as active sites. Furthermore, we comprehensively and emphatically sum up the recent advancements of carbon-based nanomaterials toward ORR from the combination of various defects and heteroatom dopants. Finally, we point out the current issues and challenges in the construction of highly efficient carbon-based materials for electrocatalysis and other electrochemical applications. This review not only provides an overview on the development of carbon-based metal-free ORR electrocatalysts, but also reveals the relationship between the defects/dopants and the electrocatalytic ORR performance.

## Heteroatom Doping

Heteroatom doping is the replacement of partial carbon atoms in the carbon skeleton by other non-metal heteroatoms (e.g., N, B, O, F, P, S, Cl, Br, I, Se), as schematically illustrated in Fig. 1b. By heteroatom doping, the properties of the carbon materials can be significantly changed in contrast to pristine ones. The introduction of heteroatoms with different size and electronegativity into the carbon matrix can induce charge and spin redistribution among the around carbon atoms, leading to the modification of electronic properties and other chemical activities for many applications [6, 18, 26, 33, 58–62].

Generally, the heteroatom-doped carbon nanomaterials can be experimentally obtained by either direct doping in the synthesis of carbon nanomaterials or post-treatment of preformed carbon nanomaterials with dopant-containing precursors [5, 51, 52]. Among all the heteroatom-doped carbon candidates, N-doped nanocarbon has been extensively studied because N atoms have similar atomic radius with C atoms, as well as possess one extra electron more than C atoms [16]. The former can prevent the lattice mismatch after inserting the heteroatom into the carbon skeleton, and the latter is in favor of the reaction that requires electron involvement like ORR [1, 13, 14, 18, 54, 62]. These two advantages endow N-doped carbons with better ORR activity and stability compared to other non-metal heteroatom-doped ones in most cases.

**Fig. 2:**
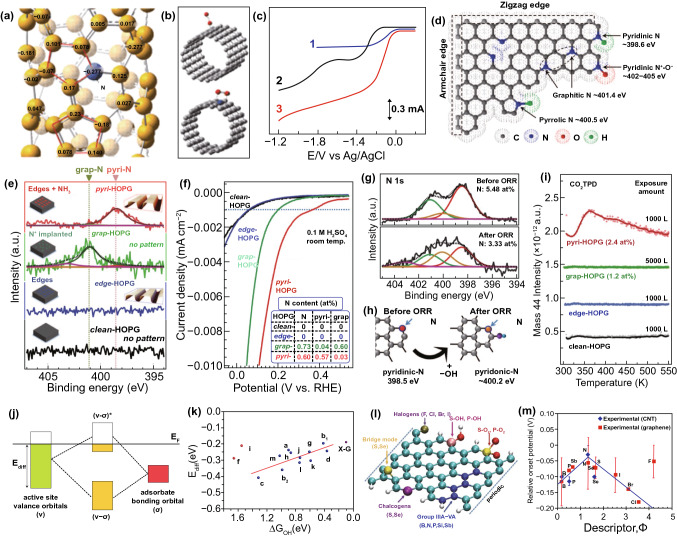
Heteroatom-doped nanocarbon catalysts for ORR. **a** Calculated charge density distribution of the N-doped CNTs; **b** schematic illustrations of two possible adsorption modes of O_2_ molecule at the pristine CNTs (top) and N-doped CNTs (bottom); **c** LSV curves for the prepared samples in air-saturated 0.1 M KOH for Pt/C (curve 1), VACCNT (curve 2), and VA-NCNT (curve 3) catalysts. Reproduced with permission from Ref. [3]. Copyright 2009, AAAS. **d** Schematic illustration of the commonly doped N species in carbon nanomaterials and the corresponded XPS binding energies. Reproduced with permission from Ref. [76]. Copyright 2015, AAAS. **e**, **f** N 1*s* XPS spectra and the corresponded LSV curves of the electrocatalysts; the inset of **f** is the N contents of the catalysts; **g** N 1*s* XPS spectra of the N-HOPG catalyst before and after ORR testing, respectively; **h** schematic illustrations of the formation of pyridinic-N by the attachment of OH to the carbon atom next to pyridinic-N. **i** CO_2_-TPD results for the four kinds of HOPG model catalysts. Reproduced with permission from Ref. [6]. Copyright 2016, AAAS; **j** scheme of orbital hybridization between the valance band of the doped carbon and the bonding orbital of adsorbates; **k** the relationship between Δ*G*_OH*_ and *E*_diff_ for various doped graphene (X–G) active sites; note that the red points were not fitted. Reproduced with permission from Ref. [81]. Copyright 2014, American Chemical Society. **l** Schematic of various heteroatoms doped graphene nanoribbons. **m** Experimentally measured relative onset potential to that of Pt/C catalyst, as a function of descriptor (*Φ*) for *p*-element-doped carbon materials. Reproduced with permission from Ref. [58]. Copyright 2015, Wiley–VCH

Pioneering work on N-doped carbon was done by Dai’s group in 2009 [3]. The prepared vertically aligned nitrogen-doped carbon nanotube (VA-NCNT, Fig. 2a, b) by the pyrolysis of iron (II) phthalocyanine precursor exhibited a higher ORR activity and longer durability without CO deactivation and fuel crossover effects compared with that of commercial Pt/C catalysts (Fig. 2c), which brings the promise to replace noble metal materials to efficiently catalyze the ORR for fuel cells. Following this pioneering work, tremendous N-doped carbon nanomaterials were developed, such as N-doped CNTs [17, 28], graphene [37, 40, 63–66], graphene nanoplatelets [37, 67, 68], graphene nanoribbons [69], graphene quantum dots [70], microporous and/or mesoporous carbon [66, 71–75]. However, the exact nature of the active sites in N-doped carbons is still a matter of debate. Usually, four N bonding configurations are located in N-doped carbon nanomaterials, including pyridinic-N, pyrrolic-N, graphitic-N (also called quaternary-N), and oxidized-N nearing of pyridinic-N, as shown in Fig. 2d [76]. Among them, pyridinic- and pyrrolic-N atoms are located at edge or defect sites; they do not increase the number of electrons in the delocalized *π*-system. Graphitic- or quaternary-N atoms replacing carbon atoms within the graphitic structure have the same configuration as carbon atoms, but they introduce extra electrons in the delocalized *π*-system [16, 18, 76]. Some researchers thought that the enhanced ORR activity can be related to the lone electron pair of pyridinic-N due to the presence of more electrons in the delocalized *π*-orbitals of the carbon framework. This is because *π*-electrons are nucleophilic reagent that can attack the oxygen molecular, endowing it with high ORR activity [16, 20]. Whereas others insisted on an opinion that graphitized N is more beneficial to ORR performance than pyridinic-N, graphitic N also provides one extra electron to the *π*-system. A recent study further suggests that both sites are synergistically responsible for the enhanced ORR activity since graphitic N increases conductivity enhancing the limited current density, and the pyridinic-N determines the onset potential of ORR [18]. For other N species (pyrrolic- and oxidized-N), most investigations indicate that they have little effect on ORR activity [17, 18, 21].

To verify the active centers of N-doped carbon nanomaterials, Guo et al. [6] precisely prepared four kinds of highly oriented pyrolytic graphite (HOPG) electrocatalysts with well-controlled *π* conjugations, which named as pyri-HOPG, grap-HOPG, edge-HOPG, and clean-HOPG, respectively, as displayed in Fig. 2e. From electrochemical linear sweep voltammetry (LSV) measurements, pyri-HOPG presents the best ORR activity in terms of the onset potential and current density (Fig. 2f), implying that pyridinic-N mainly determines the electrocatalytic ORR activity in N-doped carbons. Followed, the ex situ post-ORR X-ray photoelectron spectroscopy (XPS, Fig. 2g) results imply that OH species could react with the C atoms neighboring to pyridinic-N, meaning that pyridinic-N groups themselves are not the electrocatalytic ORR active sites, while the C atoms adjacent to pyridinic-N are active for ORR (Fig. 2h). Carbon dioxide (CO_2_) gas desorption measurements describe that only pyri-HOPG could adsorb acidic CO_2_ molecule, demonstrating that pyridinic-N species could produce the electrocatalytic sites with high activity because they can generate Lewis basic sites (Fig. 2i). This finding provides a guideline for clarification of the dopant role toward ORR reactions catalyzed by N-doped carbon nanomaterials. Following this work and the related theory, a lot of pyridinic-N-dominated [50, 77–79] or even only pyridinic-N-doped [80] nanocarbon catalysts have been made which also have shown remarkable electrocatalytic activities.

In addition to N-doping, other heteroatom dopants (i.e., B, F, P, S, Cl, Br, I, Se) have also been employed to synthesize doped carbons toward ORR. In 2014, Qiao’s group [81] investigated the ORR activity of the heteroatom-doped graphene (X–G, X = B, N, P, O, and S) catalysts. To quantitatively stand for the valence orbital level, the orbital energy difference (*E*_diff_) between the lowest active-site valence band and the highest valence band for graphene was proposed, as described in Fig. 2j. Their molecular valence orbital levels and binding strength of active atomic sites were also analyzed. According to the Δ*G*_OH*_ data and *E*_diff_ for different kinds of active sites on X–G, a linear relationship was formed (Fig. 2k). In light of this principle, the better X–G electrocatalyst with lower *E*_diff_ value and higher valence orbital levels of active atomic sites can give rise to stronger adsorption for oxygen-containing species, leading to higher ORR activity.

Recently, Xia’s group reported the principles for the design of ORR carbon materials doped by *p*-block elements including N, P, B, S, Si, Sb, Se, Cl, F, Br, I, SOH, POH, PO_2_, SO_2_, and SeO_2_, etc. [58], as schematically illustrated in Fig. 2i. They introduced a descriptor, *Φ* = (*E*_*x*_/*E*_*c*_) × (*A*_*x*_/*A*_*c*_), where *E*_*x*_ and *E*_*c*_ denote the electronegativity of the element *x* and the carbon material, respectively, and *A*_*x*_ and *A*_*c*_ are the corresponding electron affinities. They obtained the volcano relationship based on the overpotential of carbon materials doped by *p*-elements and the function of *Φ* (Fig. 2m), where nitrogen was minimum for ORR. When normalized to the benchmark ORR catalyst of Pt/C, an analogous volcano relationship between the onset potential and *Φ* was also built. They predicted that the electrocatalytic sites induced by all *p*-elements doping are C atoms adjacent to the dopants. This work demonstrates that the heteroatom doping near the edge of graphene nanoribbons is a promising strategy in the enhancement of carbon-based metal-free electrocatalyst. This view is also supported and confirmed by the N-doped carbon materials as mentioned above.

Among all the heteroatoms, N atom normally possesses higher electronegativity than C atom, which tends to receive electrons from carbon, leading to a partial positive charge on the adjacent N dopant in the carbon material [16]. On the other hand, B and P atoms tend to donate electrons to the carbon matrix, thus creating a partial positive charge on the dopant atoms [18, 75, 82, 83]. As known, the formation of partial positive and partial negative charges is in favor of the interaction and adsorption with O_2_ and the related species on the carbon-based materials [20, 21]. Strangely, S and Se atoms have a similar electronegativity to carbon, but the obtained carbon nanomaterials also describe a good ORR activity, which is attributed to the structural distortions and changes of the charge/spin densities in carbon materials [2, 22, 25, 61]. The above-mentioned results demonstrate that the electrocatalytic ORR activity of the doped carbon not only associates with the charge distribution but also with the disruption of the uniformity of the carbon matrix.

## Defect Inducing

Based on the deepening insights into the active sites of heteroatom-doped carbon nanomaterials, the defect-induced ORR catalysis mechanism has been explored. As similar to the effect of heteroatom doping, the defects in the carbon matrix can also modulate the surface state of the electronic structure (charge and spin densities) and induce the structural distortion of the carbons, thereby enhancing the electrocatalytic ORR activity. The introduced defects in the carbon skeleton may interrupt the integrity of *π* conjugation, leading to the charge polarization of carbon atoms, and generating strong adsorption to the oxygen-containing species during the ORR process [5, 9, 22, 26, 46, 55, 84–86]. Since the nanocarbons without defects do not exist in practice, exploring the effect and contribution of defects in the carbon matrix becomes crucial for developing carbon-based ORR electrocatalysts.

The defects in carbon nanomaterials can be divided into two groups: the extrinsic defects with introduced dopants (e.g., heteroatoms and/or metal atoms doping) and the intrinsic defects that are made of carbon atoms or rearrangement only, without any dopants and “foreign bodies.” Briefly, the intrinsic defects in nanocarbon include edge defects (armchair and zigzag), point defects (e.g., vacancies, holes, and voids), line defects (e.g., dislocation, grain boundaries), some of the topological defects (e.g., pentagon, heptagon, Stone–Wales defects, and their combinations), and surface defects. In this chapter, we mainly focus on the effect and relationship of various intrinsic defects in carbon nanomaterials toward ORR.

### Edge and Pore Defects

**Fig. 3:**
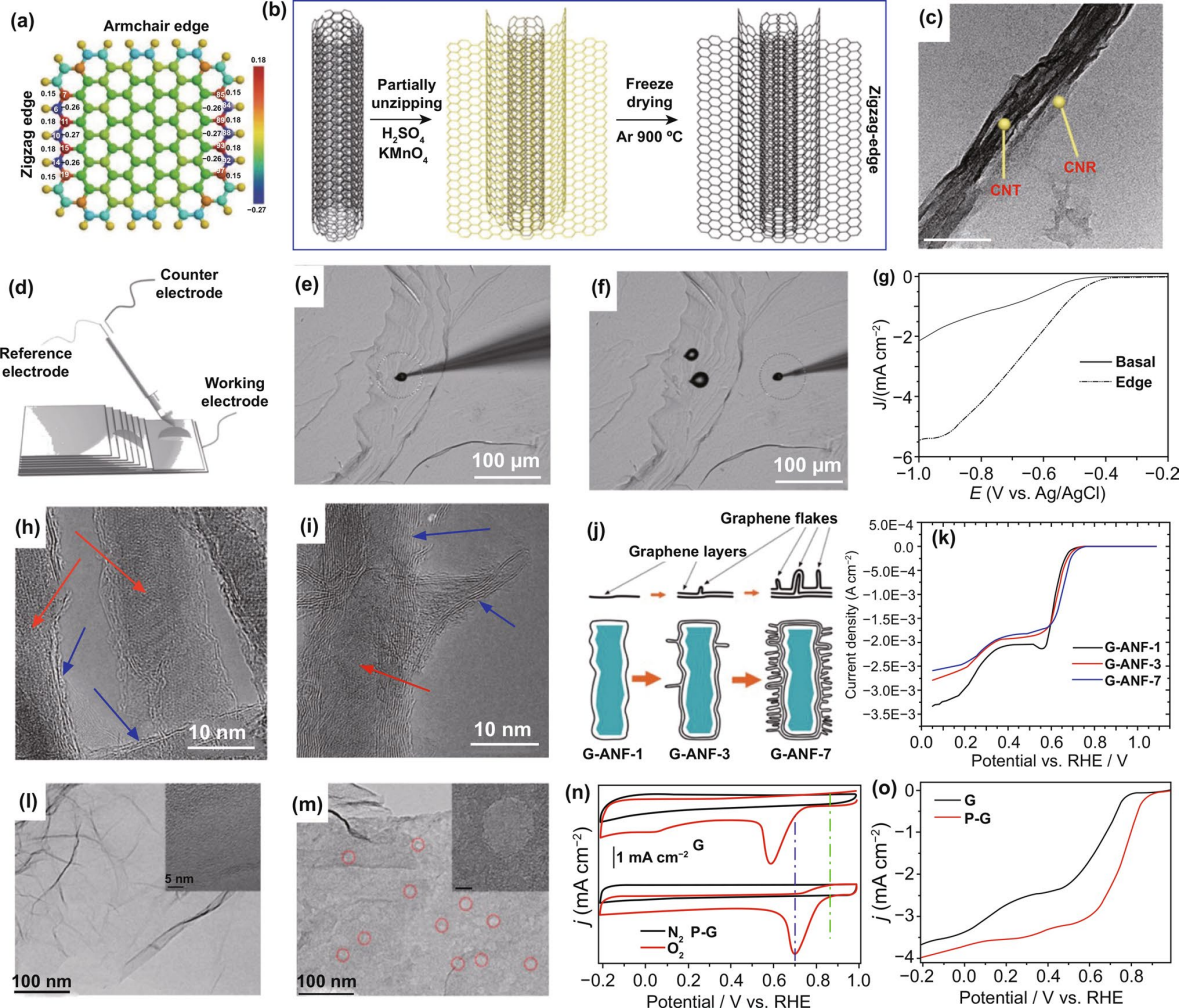
Edge- and pore-defective nanocarbon catalysts for ORR. **a** Charge and spin density distributions of graphene model. Reproduced with permission from Ref. [39]. Copyright 2015, Royal Society of Chemistry; **b** schematic illustration of the synthesis of zigzag-type graphene nanoribbons on carbon nanotubes (GNR@CNT) catalyst; **c** TEM image of GNR@CNT. Reproduced with permission from Ref. [27]. Copyright 2018, Nature Publishing Group; **d** designed micro-apparatus for ORR electrochemical measurement; **e**, **f** optical images of the working electrode (HOPG) on the edge and basal plane; **g** LSV curves for specific locations on the HOPG. Reproduced with permission from Ref. [87]. Copyright 2014, Wiley–VCH; **h**, **i** TEM images of the graphene-coated alumina nanofibers (G-ANF) by different dwell time in the CVD chamber, 20 min for G-ANF-1, 60 min for G-ANF-3; **j** schematic illustration of the series of G-ANF material synthesis; **k** LSV curves for G-ANF samples in O_2_-saturated 0.1 M KOH solution. Reproduced with permission from Ref. [63]. Copyright 2016, Royal Society of Chemistry. TEM images of pristine graphene (**l**, G) and Ar plasma-treated graphene (**m**, P–G); **n**–**o** CV and LSV curves for G and P–G catalysts in 0.1 M KOH solution. Reproduced with permission from Ref. [29]. Copyright 2016, Royal Society of Chemistry

According to different bonding states of edge sites, edges normally own various electrochemical and thermodynamic properties [51]. Armchair and zigzag edges (Fig. 3a) are the most common two defects in carbon frameworks. Based on density functional theory calculations, Xia et al. [39] investigated the electronic properties of the armchair and zigzag edge carbons using graphene cluster as the model, as described in Fig. 3a. Compared to the carbon atoms in the base plane, both armchair and zigzag edge carbons possess higher charge and spin densities, indicating that edge carbons can deliver high electrocatalytic activity since the carbon atoms with high spin/charge densities are more likely to be the catalytic active sites [29, 51, 87]. It should be also noted that the charge and spin densities of the atoms at the zigzag edge are higher than armchair ones. Because zigzag edge locations have massive unpaired *π* electrons that can greatly induce the charge and spin redistribution [51]. Therefore, the atoms in the zigzag edge have fast electron transfer and high absorption ability toward oxygen molecules and the related species.

According to this instruction, Shui et al. [27] recently synthesized the zigzag edge-enriched GNR@CNT (graphene nanoribbons coupled with carbon nanotube backbones) electrocatalyst through partially chemical unzipping the multiwalled carbon nanotubes and subsequent freeze-drying and high-temperature annealing, as schematically illustrated in Fig. 3b. The novel core–shell GNR@CNT was confirmed by the transmission electron microscope (TEM) image of Fig. 3c. Interestingly, the non-doped GNR@CNT catalyst not only exhibited a comparable ORR activity and much enhanced stability than those of the commercial Pt/C catalyst in alkaline media, but also had excellent ORR activity and stability in acidic solution, which even outperformed than those of N-doped GNR@CNT catalyst due to the immunity of protonation reaction. As a result, the as-prepared GNR@CNT catalyst showed a high peak power density of 520 W g^−1^ in acidic fuel cells, superior to the non-precious metal Fe–N–C electrocatalysts.

To directly provide the evidence to prove that the edge carbons are more active than the basal plane ones for ORR, Wang and co-workers designed a novel and classic micro-electrochemical testing system to precisely measure the ORR activity at the edge and basal plane regions, by using highly oriented pyrolytic graphite (HOPG) as the working electrode [87]. As schematically illustrated in Fig. 3d–f, a tiny electrolyte droplet was deposited on a specified location upon the HOPG surface using a micro-injection tool, so that the electrochemical signals could be collected by moving the tip to the specified location (such as the edge-rich areas or the basal plane surface). From LSV measurements of Fig. 3g, the edge carbon is more active than the basal plane, demonstrating that the edge carbon atom can be regarded as the active site for the ORR. This is the first experimental evidence that the edge carbon is more active than the basal plane, which also demonstrates the key role of the intrinsic defects for carbon-based ORR catalysts. Also, the DFT calculation results revealed that edge carbon atoms have higher charge and spin densities than the near-neutral carbon atoms [2, 15, 19, 38, 39, 51, 52, 58, 60, 88]. To further verify the edge effects for carbon-based materials, the graphite materials with abundant edges/defects were prepared by ball-milling treatment. The electrochemical measurements showed that the ORR activity was gradually improved with an increase in the defect level of the ball-milled graphite materials, as confirmed by Raman and XPS results. Following this, the ball-milled carbon nanotubes with more exposed edges also presented significantly enhanced ORR activity. This work provides a general principle for the enhancement of the ORR catalysts by edge/defect engineering.

Normally, these defect-induced carbon materials by the ball milling or chemical oxidization possess a certain oxidized surface functionalities, which may affect the electrocatalytic activity, analogous to the heteroatom doping as highlighted above. In order to research the sole effect of graphitic edges, Serban N. Stamatin et.al prepared carbon electrode materials with three different morphologies by chemical vapor deposition (CVD) technique [63]. Microscopy analysis (Fig. 3h–j) suggests that the samples are richer in graphitic edges, giving the abundant presence of foliates at the carbon surface. Electrochemical results (Fig. 3k) also imply that the ORR activity is the determination of the edge and defect density.

Pore defects in carbon nanomaterials generally refer to vacancies or holes, in which vacancy means to the absence of certain carbon atoms, and hole refers to the lack of a large range of carbon atoms, including micro-, meso-, and macropores. These absence and reconstruction features would undoubtedly break the electron–hole symmetry and possess abundant dangling groups, which would significantly modulate the local density of the *π*-electrons and increase the chemical reactivity [25]. Based on this consideration, Wang and co-workers constructed an edge-rich dopant-free graphene ORR electrocatalyst using the argon plasma etching method [29]. As shown in Fig. 3l, m, the plasma-treated graphene (P–G) possesses many nanoholes with diameters of approximately 15 nm, which can create more exposed active edge sites for ORR, giving the enhanced ORR activity (Fig. 3n, o). The electrochemical measurements implied that the ORR activity was gradually enhanced with the enhancement of the defect level. With more exposed edges and defective sites, the half-wave potential of P–G is more positive than that of pristine graphene, which can be comparable to or even better than those of doped carbon materials. Following this, the plasma-treated graphite and carbon nanotubes also exhibited improved ORR activity, indicating that plasma etching technology to create the defects is the universal strategy of the enhancement of the ORR activity in developing carbon nanomaterials. This work pushes us to consider the role of the defects and the origin of the ORR activity for metal-free carbon-based materials.

### Topological Defects

**Fig. 4:**
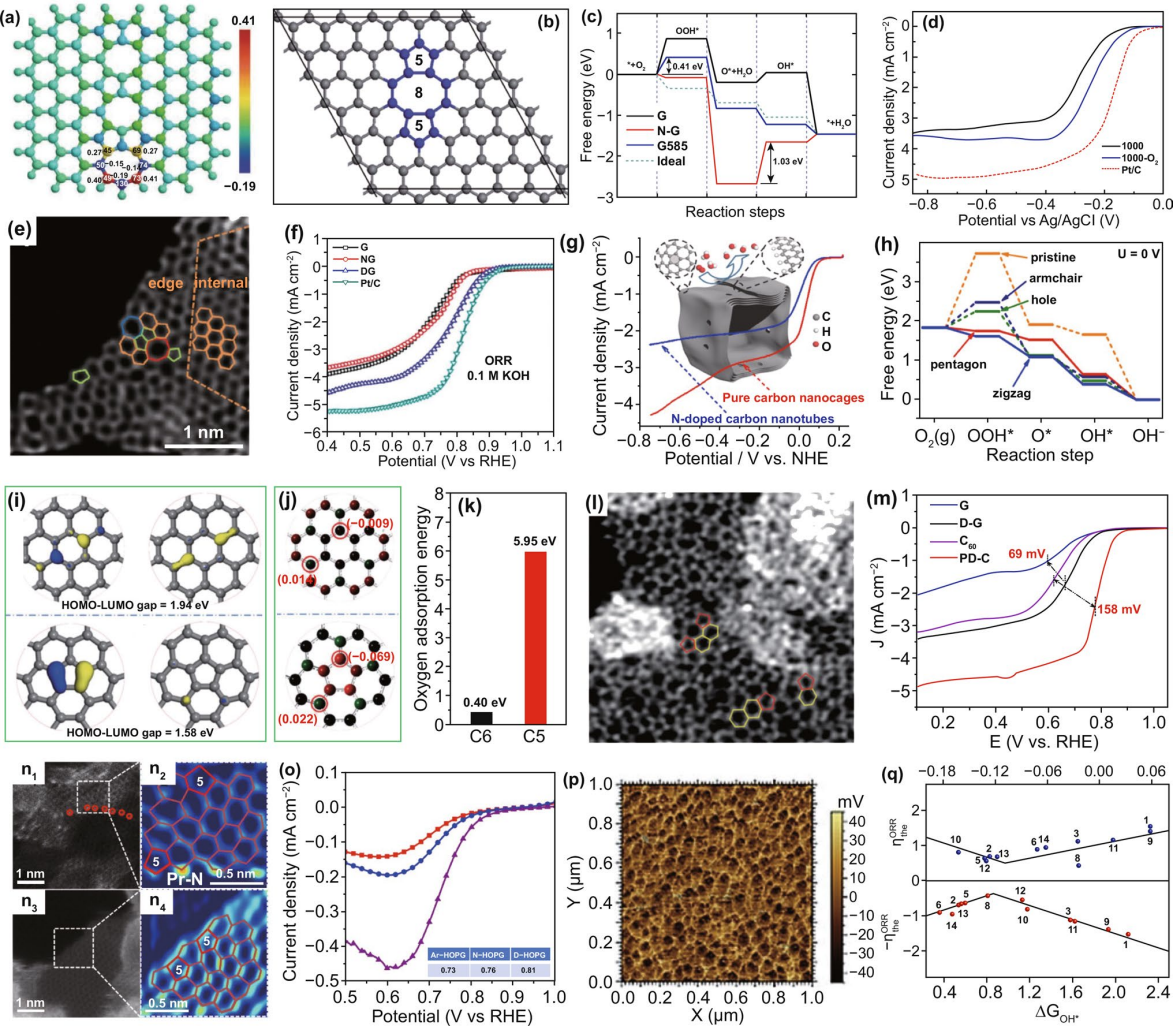
Topological defects in nanocarbon for ORR. **a** Charge and spin density distributions of the topological defective graphene model (GLD-558-01). Reproduced with permission from Ref. [39]. Copyright 2015, Royal Society of Chemistry. **b** Schematic representation of the G585 defect in graphene; **c** DFT-calculated free energy diagram of G, N–G, G585, and ideal catalysts; **d** ORR polarization curves of the prepared samples (C-1000, C-1000-O_2_) and Pt/C catalyst in an O_2_-saturated 0.1 M KOH solution. Reproduced with permission from Ref. [91]. Copyright 2015, Royal Society of Chemistry.** e** HAADF image of defective graphene (DG); **f** LSV curves for the pristine graphene, N-doped graphene (NG), DG and Pt/C catalyst in an O_2_-saturated 0.1 M KOH solution. Reproduced with permission from Ref. [40]. Copyright 2016, Wiley–VCH. **g** LSV curves of CNC700 and N-doped CNTs in O_2_-saturated 0.10 M KOH solution. The inset is the schematic structural character of CNC700 along with pentagon and zigzag edge defects; **h** DFT-calculated free energy diagrams of different defects in graphene catalyst models. Reproduced with permission from Ref. [93]. Copyright 2015, American Chemical Society. **i**–**k** HOMO–LUMO energy gap, charge density distribution, and **k** oxygen adsorption energy of pentagonal and hexagonal models. **l** STEM image of pentagon defect-rich carbon nanomaterial (PD-C); **m** LSV curves of the samples. Reproduced with permission from Ref. [92]. Copyright 2019, Wiley–VCH. **n**_**1,3**_ The HAADF–STEM images of N–G and D–G. **n**_**2,4**_ Expanded images of the dotted boxes from the corresponded N–G and D–G samples; **o** LSV curves of Ar-HOPG (red), N-HOPG (blue), and D-HOPG (purple) catalysts in 0.1 M H_2_SO_4_ solution; inset is the correlated onset potentials of the catalysts. Reproduced with permission from Ref. [45]. Copyright 2019, Nature Publishing Group. **p** KPFM test of plasma-treated HOPG; **q** The DFT-calculated overpotentials for ORR based on charge and intermediate binding energies. Reproduced with permission from Ref. [95]. Copyright 2018, Wiley–VCH

Besides the above-mentioned defects, the nanocarbon often presents some of the non-hexagonal rings (e.g., pentagon, heptagon, Stone–Wales defects, etc.) in the in-plane of the carbon material, which is also denoted as topological defects. According to Özyilmaz et al. studies [89], the topological structures are quite different from the common hexagonal carbon model, which can locally induce Gaussian curvatures and alter the bond length and angle, with rehybridized electron orbitals. These properties can also endow topological aberrant sites with local charge/spin redistribution of the carbon network (Fig. 4a), leading to the enhanced electrocatalytic activity [51, 90]. For example, Yao et al. [91] investigated the ORR performance of pentagon–octagon–pentagon defect based on the monolayer graphene model (G585, Fig. 4b). As shown in Fig. 4c, the calculated results show that G585 defect needs relatively lower activation energy for ORR than N-doped graphene and also is more favorable for the formation of OOH* from O_2_ (identified as the rate-determining step for ORR) than N-doped graphene.

More recently, Yao et al. [40] also designed defective graphene (DG) with various topological defects (pentagons, heptagons, and octagons) by removing N dopants from N-doped precursor at a high temperature. The aberration-corrected high-resolution TEM (HRTEM) image of Fig. 4e directly conforms that those topological defects are located on the edge of the graphene. Compared to pristine and N-doped graphene, the as-obtained DG reveals higher ORR onset potential and limiting current density (Fig. 4f), which can be attributed to the modulated local electronic environment and perturbed surface properties. Beyond ORR, it also presented much enhanced oxygen and hydrogen evolution reactions (OER and HER) activities. This work offers a new pathways to the construction of topological defect carbon nanomaterial for multifunctional electrocatalysis (ORR, OER, and HER).

Among all the topological defects in carbon materials, the unique pentagon defect tends to be more potential for ORR, in terms of the low reaction free energy, narrow band gap, high charge density along with high oxygen-binding ability based on DFT calculations (Fig. 4i–k) [90, 92]. In early 2015, Hu et al. synthesized carbon nanocages using magnesium oxide as a template and benzene as the precursor. They claimed that the carbon nanocages possess pentagon defects at the corners (Fig. 4g) due to the positive curvature as similar to the carbon nanotubes [93]. The theoretical calculation in Fig. 4h also proved that pentagon can substantially decrease the reaction free energy and facilitate the electron transfer for the ORR process. However, there is no direct evidence to prove the pentagon carbon rings in the presence of the carbon material. Our group successfully constructed a pentagon defect-rich carbon nanomaterial (PD–C) by directly using the pentagon-rich structure of the fullerene (C60) molecules, along with the in situ alkaline etching to unfold the molecular frameworks. As described from the aberration-corrected scanning transmission electron microscopy (ACSTEM) image of Fig. 4l, the pentagon defects were observed and reserved in the PD–C sample [92]. In contrast to the same defect degree of defective graphene catalyst, PD–C showed much more positive onset potential and larger limiting current density for ORR, indicative of the significant promoting effect of pentagon defect on electrocatalytic reactions.

Very recently, to explore the natural active sites for non-doped and N-doped carbon materials, Yao and Dai et al. [45] also fabricated the edge pentagon defect carbon materials (D-HOPG) via the high-temperature removal of the dopants from pyridinic-N-dominated HOPG. The ACSTEM images of Fig. 4n show the elimination of N atoms and the reconstruction of edge pentagon defects without the presence of dangling bonds. Compared with Ar-treated HOPG and N-doped HOPG, the as-prepared D-HOPG reveals the highest ORR onset potential in acidic media (Fig. 4o), further proving that pentagon defects play a major role of improving the ORR activity for carbon materials. According to the similar protocol of “N-doping-removal” process, a series of defective carbon-based catalysts were also reported by Hao et al. [94] using seaweed biomass sodium alginate as the precursor. The defect content, porous structure, and conductivity of defective carbons can be well controlled by tuning the heat treatment temperature and viscosity of the precursor. The optimal defective porous carbon D-PC-1(900) catalyst with large surface area of 1377 m^2^ g^−1^, abundant hierarchical porosity, and abundant ORR active defects described excellent ORR activity and high selectivity in alkaline solution. More importantly, it shows good stability and methanol tolerance for methanol in both alkaline and acidic media.

In addition to the above-mentioned edge and in-plane defects, the surface defects on carbon catalysts would also greatly influence the adsorption and desorption of intermediates associated with the ORR electrocatalysis. Based on HOPG as the research object, plasma etching technique as the defect importer, along with the Kelvin probe force microscopy and scanning ion conductance microscopy measurements, Wang and co-workers [95] investigated the relationship between the surface charge and electrocatalytic activity of the defective carbon materials. Figure 4p presents the atomic force microscope (AFM) image of the obtained HOPG sample. The extremely rough surface with plenty of hierarchical nanopores implies abundant defects in HOPG. The DFT simulations suggested that the enhancement of surface defects can improve the electrocatalytic performance including OER, HER, and ORR. A volcano relationship between surface defects and the related ORR overpotentials was obtained (Fig. 4q), which further proved that the surface intrinsic defects and charge state would significantly influence the electrocatalytic activity of carbon-based catalysts, and also might provide guidance for the rational design of defective nanomaterials with superior catalytic performance. This section is clearly indicative of the significant contribution of the intrinsic carbon defects to the electrocatalytic ORR.

## Defect and Doping Co-engineering

As mentioned above, both defect and heteroatom doping can lead to the change of the local electronic structure of carbon materials. The altered electron structures with the positive charge and/or higher charge and spin densities are beneficial for the chemisorption of oxygen molecular and the oxygen-related intermediates [2, 96], thereby contributing to the improvement in electrocatalytic ORR activity. Therefore, connecting together with various defects and heteroatom dopants or designing heteroatom dopants at specific defective sites is the most effective strategy to modify electron structures and obtain optimal electrocatalytic ORR performance for metal-free carbon-based electrocatalysts.

### Edge Defects Coupled with Dopants

**Fig. 5:**
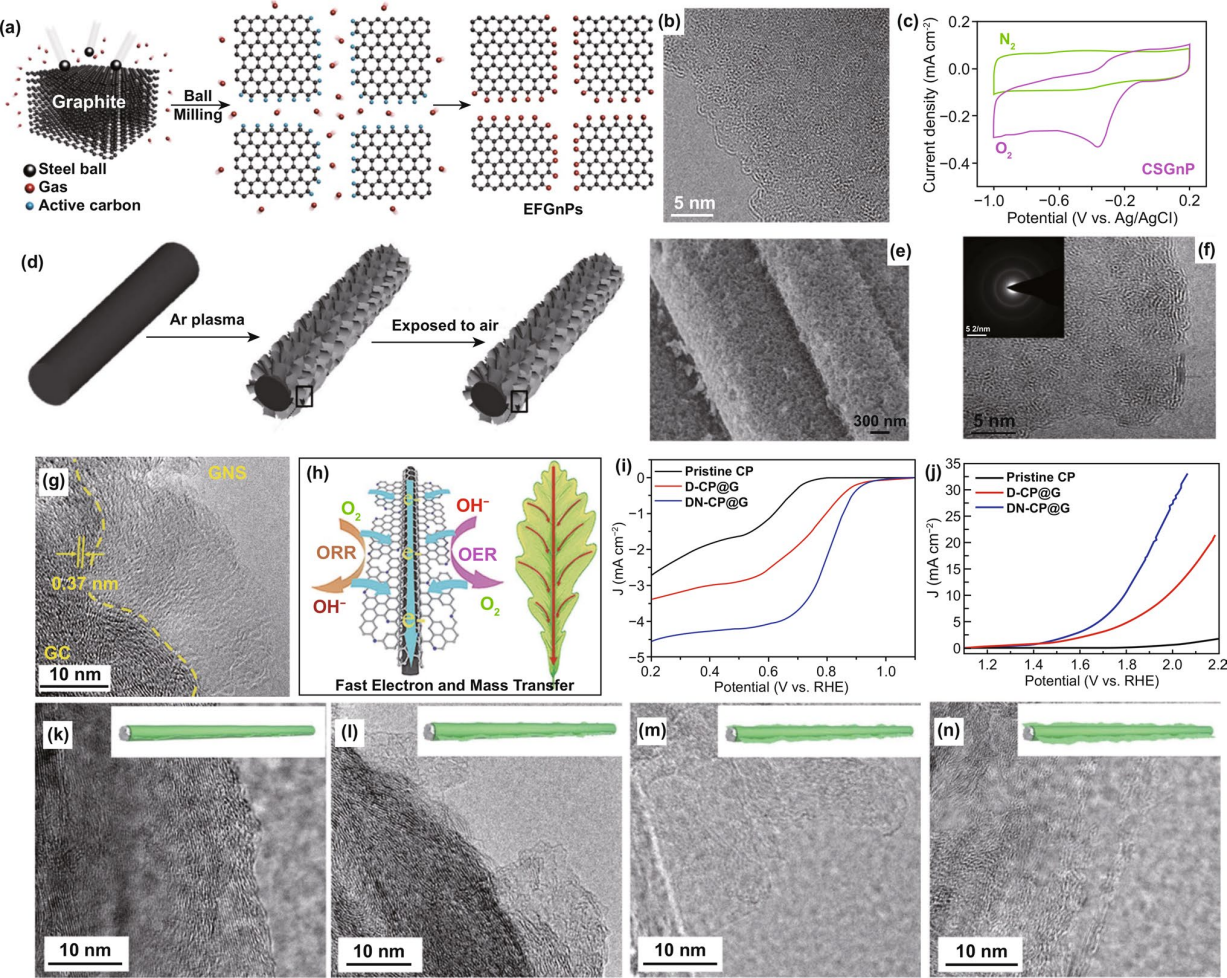
Edge defects coupled with dopants in nanocarbon for ORR. **a** Schematic representation of edge-functionalized graphene nanoplatelets (EFGnPs); **b** TEM image of EFGnPs; **c** CV curves for CSGnP catalyst. Reproduced with permission from Ref. [67]. Copyright 2013, American Chemical Society. **d** Schematic representation of edge-rich and oxygen-functionalized graphene from carbon fiber. **e**, **f** TEM image of DN-CP@G. Reproduced with permission from Ref. [64]. Copyright 2014, Wiley–VCH. **g** TEM image of DN-CP@G; **h** schematic representation of the electrocatalytic characteristic of DN-CP@G; **i**, **j** ORR and OER activities of pristine CP, and non-doped D-CP@G and DN-CP@G catalysts; **k**–**n** TEM images of CP-based materials with controlled reaction time from 4 to 16 h. Reproduced with permission from Ref. [156]. Copyright 2014, Wiley–VCH

The edge defects commonly exist in all the carbon-based materials. Compared to the in-plane carbon atoms, the edge carbon atoms are more likely to be doped or functionalized with heteroatom dopants [60, 97, 98]. Dai’s group [67] developed an edge-selectively functionalized graphene nanoplatelets as ORR electrocatalyst by simply dry ball-milling of graphite precursor in the presence of the reactant gases (Fig. 5a). The TEM image shows the ball-milled sample with plenty of folding and wrinkles (Fig. 5b), indicative of the existence of a large number of edge defects and edge functional groups, as also confirmed by XRD, XPS, and Raman results. They found that the edge polar nature of the graphite-based materials played a crucial part in modulating the ORR activity. As described in the cyclic voltammetry (CV) curve of Fig. 5c, the edge sulfur-functionalized CSGnP catalyst displays a large ORR peak area with a highly positive onset potential. Following this, they used the same method to prepare various edge-selectively halogenated (Cl, Br, and I) graphene nanoplatelets [68]. Compared to pristine graphite, the prepared three edge-halogenated graphene catalysts exhibited enhanced ORR performance. In addition to the ball milling method, the use of plasma treatment is another powerful technique to generate edge-rich carbon nanomaterials. Wang and Xia et al. [64] prepared edge oxygen-functionalized graphene by the Ar plasma treatment of carbon cloth and then exposed in the air (Fig. 5d–f). Stemming from the synergistic effect of the exposed edges and oxygen-functionalized groups, the as-obtained carbon cloth displayed admirable electrocatalytic performances for both ORR and OER. Similarly, Zhang et al. [99] developed a coaxial cable-like catalyst with carbon fiber skeleton coated by defect-rich graphene skin by using carbon cloth as substrate material along with an in situ H_2_ etching method. Thanks to abundant heteroatoms and defects in the outer graphene skin, the obtained o-CC–H_2_ catalyst exhibited 20 and 3 times current densities for OER and ORR than those of pristine carbon cloth, respectively.

Inspired by the main electrocatalytic active N dopant of pyridinic-N along with the edge defect promoting effect, our group constructed a defect-enriched and pyridinic-N-dominated bifunctional electrode with a novel core–shell architecture by in situ exfoliating and generating active sites on graphene nanosheets strongly coupled with carbon fiber (DN-CP@G) [77]. As exhibited in Fig. 5g, the shell of graphene has abundant defective sites and active pyridinic-N dopants (as confirmed by Raman and XPS results, respectively), which is responsible to generate highly effective active sites for electrocatalytic reactions. The graphitic carbon fiber core provides high conductivity with an intrinsic 3D porous structure, which enables the fast electron and mass transfer during the reactions. Such novel architecture is similar to the photosynthetic plants (Fig. 5h) in which all the leaves (active graphene nanosheets) serve as the reaction place by contacting with light and gas, and the stem (graphitic carbon fiber) is in charge of transferring the reactants. As a result, the obtained DN-CP@G sample reveals much enhanced bifunctional ORR and OER performance (Fig. 5i, j) under three-electrode system compared to pristine CP (carbon paper) and non-doped D-CP@G samples. To further investigate the relationship between the electrocatalytic activities (including ORR and OER) and the defects/dopants, the different defect degree and N-doped content of CP-based materials were well controlled by tuning the exfoliation time. As shown in TEM images of Fig. 5k–n, the outer layer of defective graphene nanosheets becomes larger and thinner with the extension of reaction time from 4 to 16 h. And also, Raman and XPS spectra results showed that both defect degree (in terms of the increased intensity ratio of the D band to G band) and N-doped content including active pyridinic-N dopant in these CP-based materials gradually increase. The electrocatalytic activities (ORR and OER) of the corresponding samples also increased in turn. We then summarized and analyzed the critical characteristics (including defect degree and active dopant content) of these CP-based materials along with their key parameters of ORR and OER. It was concluded that their electrocatalytic activities strongly associate with their defective sites and active N dopants. Similarly, Yin and co-workers [78] reported laser-induced pyridinic-N-rich defective carbon nanotubes (CNTs). The laser irradiation not only can introduce a lot of edge structure and promote the N-doping with the dominated pyridinic-N content, but also can improve the specific surface area and pore density of CNTs. The ORR half-wave potential of the optimized NL-CNT-3 catalyst reached up to 0.84 V, which was much higher than those of pristine CNT and the direct N-doped CNT (fewer defects) and the undoped defective CNT. These results strongly suggest that the importance of cooperation with the edge and edge-functionalized groups in the development of the high-efficient nanocarbon electrocatalysts.

### Pore Defects Coupled with Dopants

Fabrication of porous structure including micropore, mesopore, macropore and hierarchical porous structure is another promising strategy to enhance the electrocatalytic activities for nanocarbon materials. Strictly speaking, the pore or hole belongs to a specific structure category in carbon-based materials, but at the same time, it could bring a lot of defects such as vacancies, voids, abundant edges, non-hexagonal topological defects (e. g., pentagon, heptagon, and Stone–Wales defects) on the corner or curvature places [93]. As mentioned above, these defects in carbon-based materials could break the electron–hole symmetry, significantly alter the charge and spin distributions, as well as tune the binding affinity surface adsorption/desorption behaviors of the oxygen-related intermediates [25, 100, 101]. Beyond these benefits, the porous structure with enhanced specific surface area could maximize the exposure of the active site with high utilization and facilitate the mass and charge transportation during the ORR [35, 71, 72, 100, 102–105]. Therefore, many heteroatom-doped porous nanostructured carbon-based materials with improved electrochemical properties have been well developed in recent years.

**Fig. 6:**
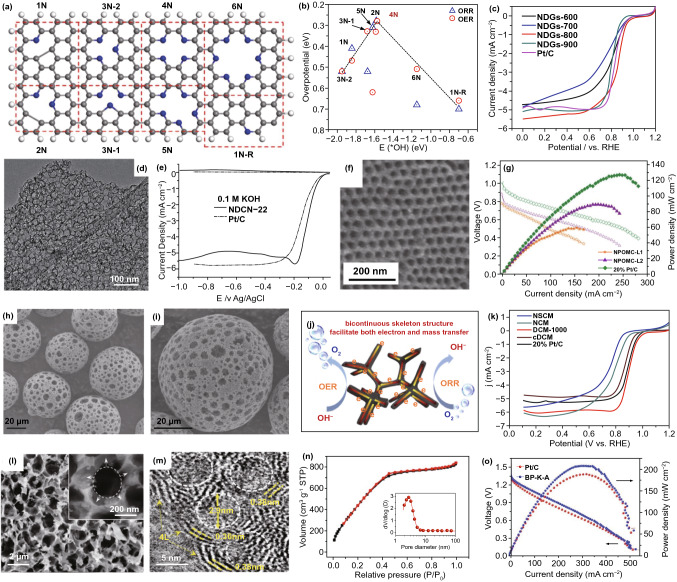
Pore defects coupled with dopants in nanocarbon for ORR. **a** Synergetic active sites of vacancy defects and N dopants, and **b** the corresponding ORR and OER volcano plots; **c** ORR polarization curves of the samples and Pt/C catalyst. Reproduced with permission from Ref. [50]. Copyright 2018, American Chemical Society. **d** TEM image of NDCN-22; **e** RRDE measurement of NDCN-22 and Pt/C catalysts in alkaline media. Reproduced with permission from Ref. [106]. Copyright 2014, Wiley–VCH. **f** TEM image of NPOMC-L2 sample; **g** polarization curves of AEMFCs used NPOMC-L1, NPOMC-L2, and Pt/C catalysts. Reproduced with permission from Ref. [107]. Copyright 2015, Wiley–VCH. **h**, **i** SEM images of DCM-1000; **j** schematic representation of DCM-1000 for ORR and OER; **k** LSV curves of the samples and Pt/C catalyst. Reproduced with permission from Ref. [109]. Copyright 2019, Elsevier. **l** SEM and **m** TEM images of BP–K–A; **n** N_2_ adsorption–desorption isotherm and pore size distribution of BP–K–A; **o** Polarization curves and power density plots of Zn–air batteries fabricated with BP–K–A and Pt/C electrodes. Reproduced with permission from Ref. [110]. Copyright 2017, Elsevier

Vacancy, as one of the micropore defects, means to the lack of a range of carbon atoms, which commonly exists in most carbon-based materials. Lei and co-workers investigated the ORR and OER catalytic sites of vacancy defect-enriched and pyridinic-N-dominated graphene (NDG) based on DFT calculations (Fig. 6a) [50]. It suggested that the quadri-pyridinic-N configuration at the edge of vacancy defect (4 N) owns the lowest overpotential for both ORR and OER (Fig. 6b). It has a fast charge transfer tendency with low HOMO–LUMO energy band gap, indicative of the collaborative mechanism of vacancy defect and N dopant. The as-prepared NDGs-800 aerogel with ultrahigh pore volume of 3.43 cm^3^ g^−1^ displays excellent ORR activity with a half-wave potential of 0.85 V in 0.1 M KOH solution (Fig. 6c).

Mesopore also facilitates to the ORR due to the enhanced mass transportation of ORR-relevant species, large pore volume, and high specific surface area [74, 100, 106, 107]. Mullen and Feng et al. well designed uniform mesoporous N-doped carbon nanosheets (NDCNs, Fig. 6d) with graphene-like structure by using nanosilica template approach [106]. The mesoporous size of NOCNs can be controlled by the regulation of the silica template. As a result, the NDCN sample with the pore size of 22 nm (NDCN-22, Fig. 6e) exhibits the best ORR performance in alkaline media, as compared to that of Pt/C. In addition, Lee et al. [107] investigated the effect of the pore size and heteroatom-doped site position for the carbon-based metal-free catalysts using well-defined ordered mesoporous carbon material. The as-synthesized N-, P-codoped ordered mesoporous carbon with large pore size (NPOMC-L2, Fig. 6f) catalyst shows a remarkable ORR onset potential on RDE measurement with 70% of the maximum power density of Pt/C electrode in single-cell alkaline anion exchange membrane fuel cells (AEMFCs) test at the operation of 60 ℃ (Fig. 6g). Recently, Chen’s group also demonstrated the mesopore-dominated N-doped carbon framework (NDCF) catalyst that owns higher ORR activity than those of non-porous samples [108].

Macropores can maximize and ensure the acceleration of the transformation of O_2_ bubbles and their related species [108–113]. For example, Zhou et al. [109] fabricated a defect-enriched N-doped graphitic carbon sphere with fully opened and 3D-interconnected supermacroporous (Fig. 6h–k) oxygen bifunctional (ORR and OER) catalyst by the pyrolysis of the macroporous polystyrene sphere template coated with the combination of dopamine and cysteamine, and ammonia injection under the high temperature. The obtained supermacroporous carbon material not only features continuous porous structures with high surface area, but also possesses abundant active defective sites and N dopants, ensuring it with excellent bifunctional electrocatalytic performances toward OER/ORR, as well as corresponding rechargeable zinc–air batteries.

From the above, all kinds of pores have their own unique advantages for electrocatalysis. Hence, developing hierarchically porous nanostructure in carbon-based electrocatalyst is highly desirable [71–73, 111, 114–116]. By employing natural porous banana peel as precursor along with KOH activation, we developed a defective N-doped graphene-like nanosheets ORR catalyst that owns extremely high specific surface area (1756 m^2^ g^−1^) and 3D interconnected hierarchical porous structure [110], as confirmed by the SEM and TEM images and N_2_ adsorption–desorption isotherm and pore size distribution results (Fig. 6l–n). Remarkably, such hierarchical porous graphene-like (BP–K–A) catalyst not only exhibits an outperformed ORR activity than commercial Pt/C in 0.1 M KOH electrolyte, but also has a higher power density than Pt/C in real zinc–air battery (Fig. 6o). This could be attributed to the cooperative effect of ultrahigh surface area, excellent porous structure, enhanced electronic conductivity, rich edge defects, as well as high active nitrogen-doped content. Similarly, Wang et al. [114] obtained a 3D honeycombed hierarchical porous N and O dual doped carbon (HHPC) via the combination strategy of CaCO_3_ hard template and KHCO_3_ activator-assisted pyrolysis. The unique hierarchically macro–meso–microporous framework and N-, O-codoped features made HHPC as the outstanding bifunctional (ORR and OER) activity. Jiang et al. [117] synthesized graphene-like and defect-rich N-doped carbon nanosheets (GPNCS) electrocatalyst via a hydrothermal reaction, synchronous KHCO_3_ carbonization and activation, and N-doping process. The GPNCS material not only features graphene-like morphology with few layers and interconnected hierarchically porous structure, but also has abundant defective structures including edges, pores, forks, and cracks, which endow it with remarkable ORR and OER performance. These studies suggest that the adjustment of the pore defect in nanocarbons is another viable option to optimize the catalytic activity.

### Topological Defects Coupled with Dopants

**Fig. 7:**
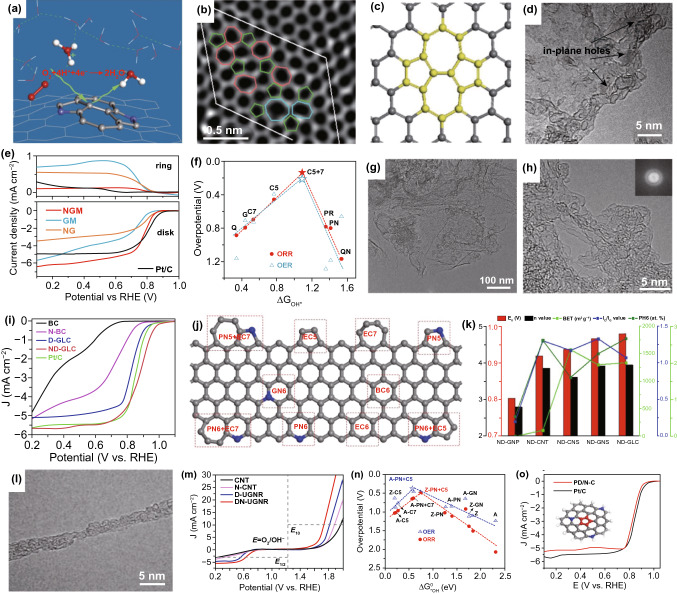
Topological defects coupled with dopants in nanocarbon for ORR. **a** Schematic structure of N-pair-doped Stone–Wales defect. Reproduced with permission from Ref. [118] Copyright 2014, American Chemical Society. **b** TEM image of single-layer defective graphene; **c** schematic of pyrrolic N-doped graphene with topological defects. Reproduced with permission from Ref. [121]. Copyright 2016, Wiley–VCH. **d** TEM image of NGM sample; **e** RRDE measurements of the prepared samples and Pt/C catalyst in O_2_-saturated 0.10 M KOH; **f** DFT-calculated volcano plots of overpotential versus △GOH* based on various N dopants and topological defects. Reproduced with permission from Ref. [30]. Copyright 2016, Wiley–VCH. **g**, **h** TEM images of ND-GLC; **i** LSV curves of BC, N-BC, D-GLC, ND-GLC, and Pt/C catalysts; **j** proposed active sites for ORR based on graphene models; **k** ORR activities (*E*_0_ and *n* value) of the catalysts along with their critical characteristics (BET surface area, *I*_D_/*I*_G_ value, and the content of PN6 dopant). Reproduced with permission from Ref. [77]. Copyright 2018, Elsevier. **l** TEM image of DN-UGNR; **m** ORR and OER polarization curves for various samples; **n** DFT-calculated volcano plots for ORR and OER. **o** ORR polarization curves for PD/N–C and Pt/C catalysts. Reproduced with permission from Ref. [92]. Copyright 2019, Wiley–VCH

The non-hexagonal structure of topological defects can also cooperate with heteroatom dopants to significantly promote the electrocatalytic activities. Based on the first-principles calculations, Chai and co-workers investigated the possible ORR active sites and mechanism on N-doped carbon alloy catalysts [118]. They found that a particular structure of N-pair-doped Stone–Wales defect (Fig. 7a) can provide good ORR activity with high limiting potential (ca. 0.80 V) owing to the tuned curvature effect [2, 25]. As known, the high electrical double-layer (EDL) capacitance in nanocarbons can bring high concentration of active sites for ORR; thereafter, the ORR performance is related to the EDL of carbon materials [119, 120]. By using single-layer graphene as the electrode [121], Ji et al. investigated the role of topological defects and N dopants (Fig. 7b) on the EDL capacitance. They found both topological defects and N dopants can improve the EDL capacitance of the carbon-based materials, in which topological defects enhance the density of states (DOS) and the N dopants modulate the Fermi level of graphene.

Likewise, Zhang’s group demonstrated the critical contribution from topological defects of N-doped graphene on the electrocatalytic activity [30]. The N-doped graphene mesh (NGM, Fig. 7d) with abundant edges and in-plane nanopores was prepared by directly carbonizing carbon and nitrogen precursors on MgO templates. The as-obtained NGM catalyst exhibits much enhanced ORR activity in contrast to the counterpart that only has topological defects or N dopants, as revealed in Fig. 7e. They established the models including N dopants and some topological defects, to predict the correlation between oxygen adsorption energy of OH with OER and ORR activity. It was found that topological pentagon–heptagon pair (C5 + 7) and pentagon (C5) are superior to all nitrogen species for the oxygen intermediates adsorption, as described in Fig. 7f. The key intermediates of electrochemical reactions tend to be adsorbed on the junction of the optimal C5 + 7 mode. Even so, in case of the high content of N dopants (≈ 7.60 at.%) in NGM, we consider that the excellent ORR activity of NGM should be originated from the synergistic effect of topological defects and N dopants.

For the co-construction of topological defects and specific heteroatom dopants, we designed a pyridinic-N-dominated and defect-enriched graphene-like carbon nanomaterial (ND-GLC, Fig. 7g, h) via a facile and general strategy involving in situ alkaline activation of cellulose and ammonia injection [77]. In contrast to non-doped defective GLC (D-GLC) and directly ammonia-treated bulk carbon (N-BC), the ND-GLC material exhibits the best ORR performance (Fig. 7i). Experimental and theoretical studies revealed that the excellent catalytic activity of ND-GLC mainly originates from the synthetic promoting effect of edges/defects and pyridinic-N dopant. As an example, the pyridinic-N adjacent to edge pentagon defect (PN6 + EC5, Fig. 7j) site predicts the lowest binding energy of key intermediate of OH among all the possible active graphene models. Importantly, our concept demonstrated to be universal for other carbon-based nanomaterials such as graphite nanoplates, carbon nanotubes, carbon nanospheres, and graphene nanosheets. The significant outcome of the systematical analysis implies that the more the edges/defects and pyridinic-N dopants in the carbon matrix (Fig. 7k), the higher the electrocatalytic activities are.

As described above, the topological defect of EC5 in nanocarbon is of great important for ORR. Very recently, Yao and co-workers [122] used the DFT simulation to investigate the influence of various heteroatom dopants on the EC5 defective graphene for ORR. The results revealed that the N- and S-codoped defective graphene with edge C5 defect possesses the smallest ORR overpotential among all the established models. Although they claim that the heteroatom dopants have limited contributions to the ORR, the defect sites such as EC5 are essential for catalyzing the ORR. Undeniably, they also agree that the heteroatom dopants are powerful in tuning the electronic properties of defective sites for the enhancement of ORR activity, further indicative of the synergistic promoting effect of the nanocarbon ORR electrocatalyst by defect and doping co-engineering.

Furthermore, considering abundant topological defects (e.g., pentagons and heptagons) in the presence of carbon nanotubes due to the local curvatures [17, 77], we developed a topological defective N-doped ultranarrow graphene nanoribbons (DN-UGNR, Fig. 7l) by chemical oxidation and unzipping of carbon nanotubes (CNTs) along with ammonia treatment [69]. It shows both high ORR and OER activities in alkaline condition (Fig. 7m), demonstrating that the co-engineered defect and N dopant in carbon materials can greatly improve the electrocatalytic activities. To unveil the reasons behind these phenomena, the volcano plots for ORR and OER are constructed in Fig. 7n. Based on the results, we found that these key intermediate species tend to be stable on the adjacent pyridinic-N and pentagon carbon rings. This synergistic strategy by coupling with topological defects and N dopants for promoting electrocatalytic ORR activity was also demonstrated by our recent work used of N-doping into pentagon defect-enriched nanocarbons **(**Fig. 7o) [92]. These nanocarbon materials not only showed a good ORR activity in both RDE measurements and the practical zinc–air batteries, but also possessed an outstanding EDL capacitance. Based on the mentioned above, the electrocatalytic activities including ORR and OER can be tuned and boosted by the combination of topological defects (especially for pentagon defect) and heteroatom dopants.

### Defects Coupled with Multi Dopants

**Fig. 8:**
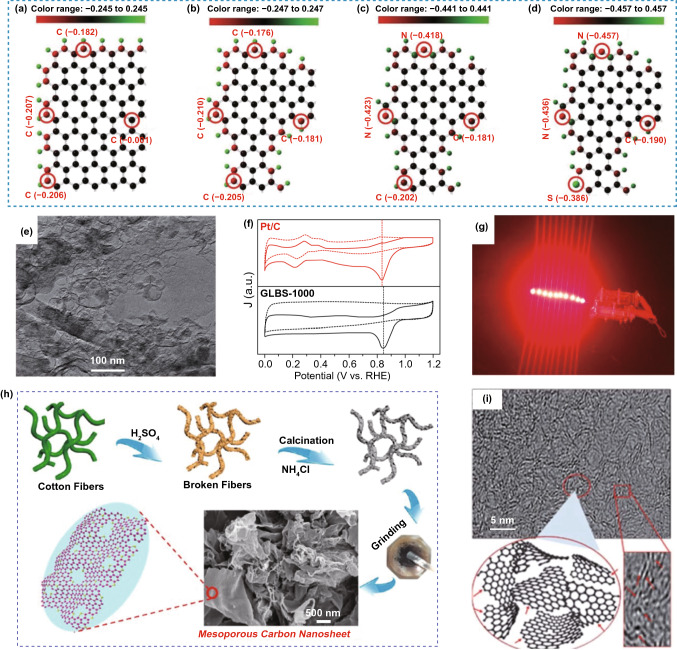
Defects coupled with multiple dopants in nanocarbon for ORR. **a**–**d** Charge density distribution of pristine, defective, N-doped defective and N/S-codoped defective graphene models. Reproduced with permission from Ref. [112] Copyright 2018, Wiley–VCH. **e** TEM image of GLBS-1000 sample with the hybridized graphene-like nanobubble and nanosheet architectures; **f** CV curves for GLBS-1000 and Pt/C catalysts. Reproduced with permission from Ref. [102]. Copyright 2016, The Royal Society of Chemistry. **g** Photograph of 10 parallel red LED lamp beads driven by two Zn–air batteries with the D-S/N-GLC electrode connected in series. Reproduced with permission from Ref. [128]. Copyright 2017, American Chemical Society. **h** Schematic of cotton microfiber-derived mesoporous N-doped carbon catalyst. Reproduced with permission from Ref. [132] Copyright 2017, American Chemical Society. **i** TEM image of PC and schematic illustration of the defective structure. Reproduced with permission from Ref. [133]. Copyright 2017, The Royal Society of Chemistry

As reported, the ORR performance of the carbon-based materials can be further improved by dual- and multi-doping of heteroatoms (e.g., N/B; N/S; N/P; O/N, P/O, N/S, B/S/N, and P/N/S) due to their synergistic effects [123–126]. In a similar way, the combination of various defects with dual- and multi-heteroatom doping also provides powerful means for the possible of maximum enhancement of various electrocatalytic performances for carbon nanomaterials. By sequential comparison, Mu and co-workers [51, 112] investigated the charge density distribution of four graphene-based models including pristine graphene, defected graphene, N-doped defected graphene, and N/S-codoped defected graphene (NS–D–G), as schematically shown in Fig. 8a–d. The carbon atoms located on the edges, holes, or adjacent to heteroatom (N/S) dopants reveal the higher charge densities compared to those of pristine graphene, indicative of the higher ORR activity based on Xia and co-workers’ prediction [39, 64, 127]. Furthermore, arising from the synergistic effect of the intrinsic defects and S/N dopants, the NS–D–G model exhibited the smallest HOMO–LUMO energy gap among them, indicative of the fast electron transfer capability, which can be conducive to electrochemical reactions. Experimentally, the fabricated defect-rich N/S-codoped cheese-like porous material displayed considerable ORR activity in contrast to the conventional Pt/C catalyst in both RDE measurements and the primary zinc–air batteries.

They also developed an S- and N-codoped graphene-like nanobubble and nanosheet hybridized architecture electrocatalyst (Fig. 8e) using keratin as a precursor, subsequently pre-carbonization, KOH activation, and high-temperature ammonia treatment [102]. During the high-temperature KOH activation, the produced metallic K atoms can be intercalated and diffused into the carbon layer, expanding the graphite layer spacing to form graphene-like carbons. And the violent corrosion processes would lead to the formation of abundant edges and defects in the product [102, 110, 128, 129]. Benefiting from the novel nanobubble and nanosheet hybridized architecture, superhigh specific surface area, hierarchical porous structures, and the dual effect of S/N dopants and abundant active defect sites, the ORR onset potential of the prepared material is superior to that of benchmark Pt/C catalyst (Fig. 8f). Furthermore, a similar protocol was employed to prepare a defect-enriched and dual heteroatom (S and N)-doped hierarchically porous graphene-like carbon (D–S/N-GLC) electrocatalyst by using cysteine as a precursor [128]. The D–S/N-GLC has a similar morphology of graphene, superhigh specific surface area with excellent 3D hierarchically porous structures, high content of active heteroatom dopants, as well as abundant defective sites, endowing it with outperformed ORR activity and Zn–air battery performance (Fig. 8g).

It should be here noted that the conversion of biomass materials such as keratin and cysteine into the functionalized carbon nanomaterials with defect-rich and heteroatom dopants for ORR has attracted extensive attention due to their self-enriched heteroatoms (e.g., N, P, S), renewable resources, abundant, convenient operation process, and environmental benignity [130, 131]. For example, Tian et al. took absorbent cotton microfibers as raw material, pre-impregnated with fuming sulfuric acid, and thermally annealed (Fig. 8h), and obtained N-doped thin carbon nanosheets with a large number of mesoporous as well as a considerable nitrogen content of 8.5 at.% [132]. These characteristics contribute together to excellent ORR activity. Kim et.al reported a porous carbon (PC) catalyst with defects and self-doped oxygen functional groups from grapefruit peel biowaste using hydrothermal carbonization coupled with chemical activation [133]. TEM image (Fig. 8i) displays the disordered aromatic structure and the twisted edge (zigzag and armchair edges) of the graphene layer in PC product. They suggested that the spin-polarized carbon edges functionalized with functional groups or heteroatom dopant usually showed higher electrocatalytic activities than non-spin-polarized edges due to the altered charge and spin distributions. Except for these examples, various plant and animal biomass materials such as eggs [134], eggplant [135], soybean shells [136], sheep wools [71], Typha orientalis [137], sheep horn [138], human hair [139], chitosan [140], cellulose [141], glucose- and lignin-based materials [130, 142] have been chosen as raw materials to obtain the defective and heteroatom-doped carbon nanomaterials. The results also demonstrate the synergistic effect of topological defects and multi-heteroatom dopants to boost the electrochemical performance for carbon-based materials.

**Fig. 9:**
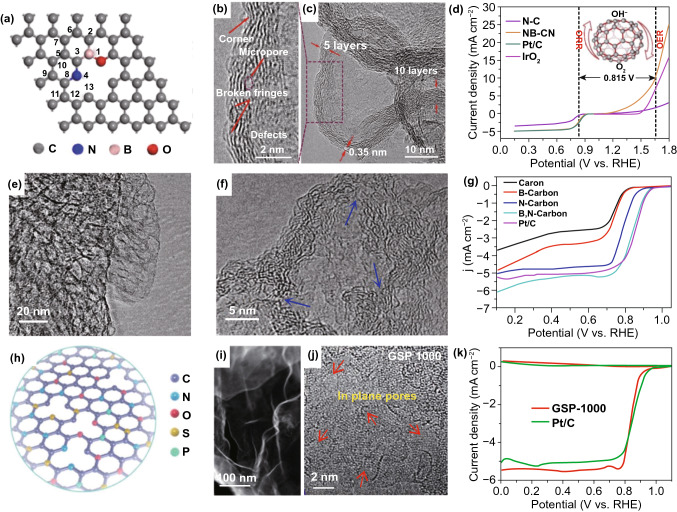
Defects coupled with multiple dopants in nanocarbon for ORR. **a** Schematic illustration of B-/N-codoped defective nanocarbon; **b**, **c** TEM images of B-/N-codoped carbon with various defective sites; **d** ORR and OER performance of NB-CN. Reproduced with permission from Ref. [143]. Copyright 2017, Elsevier. **e, f** TEM images of B-/N-codoped carbon hollow nanocages; **g** ORR activity of various samples. Reproduced with permission from Ref. [144]. Copyright 2018, Wiley–VCH. **h** Schematic illustration of multiple heteroatom-doped (N/S/P/O) nanocarbon; **i**, **j** TEM images of N/O/S/P-doped carbon containing a large number of in-plane pore defects; **k** RRDE measurements of GSP-1000 and Pt/C catalysts. Reproduced with permission from Ref. [147]. Copyright 2018, The Royal Society of Chemistry

The defect engineering coupled with B, N codoping is another efficient strategy for the development and enhancement of catalytic activities for nanocarbons. Very recently, Zhao et al. [143] fabricated a N, B-codoped graphitic carbon nanocage (NB-CN, Fig. 9a–c) with graphitic defect-rich characteristic trifunctional (ORR, OER, and HER) electrocatalyst. DFT calculations demonstrated that the excellent electrocatalytic activities originate from the configuration with B and pyridinic-N dopants on the edges, which presents the minimum theoretical overpotential toward ORR and OER, as well as the lowest Gibbs free energy for HER. Figure 9d delivers ORR and OER polarization curves for NB-CN catalyst that experimentally demonstrate excellent bifunctional activities. By pyrolyzing the composites of ethyl cellulose and high boiling point 4-(1-naphthyl) benzene boronic acid in NH_3_ atmosphere, Su group [144] also prepared B-, N-codoped and defect-rich nanocarbon with interconnected cuboidal hollow nanocages (Fig. 9e–g) that performed high ORR activity with an onset potential reaching 0.98 V and excellent OER activity approaching to that of precious metal RuO_2_ catalyst, as well as low voltage gap between charge and discharge and long-term stability in zinc–air batteries. The authors demonstrated that the outstanding electrocatalytic performance of this material was attributed to the combined positive effects of heteroatom (B and N) co-dopants and abundant carbon defects.

Based on edge-defective B-/N-codoped graphene nanoribbon model and the spin-charge density distribution and local density of state for O_2_ adsorption [145], Zou et al. found that the adsorbed O_2_ molecular displayed large spin splitting and broadening of interacting states, indicative of stronger binding of O_2_. Chattopadhyay et al. also found that B/N dual doping with defect could modulate the band gap of graphene, which could significantly stretch the O–O bond, decreasing the bond breakage barrier for oxygen reduction [146]. Xie and co-workers developed a series of multiple heteroatoms (N/O, N/S/O, N/P/O, and N/S/P/O, Fig. 9h)-doped nanocarbons by the pyrolysis of biomolecule guanine aggregates and/or the obtained salts ionized with the concentrated acid H_2_SO_4_ and H_3_PO_4_ [147]. As seen from the TEM images in Fig. 9i, j, the obtained GSP-1000 displays a larger number of disordered lattice fringes with in-plane pore defects on the surface. Due to the multiple heteroatom doping and doping-induced porous structure and defects, the optimized GSP-1000 catalyst shows remarkably enhanced ORR activity compared to the counterparts with less heteroatom dopants and/or defects. RRDE data in Fig. 9k show that GSP-1000 catalyst has a similar ORR kinetics and selectivity to Pt/C catalyst. Interestingly, it also shows a high ORR activity under universal pH conditions including alkaline, neutral, and acid media. Inspired by this, Pham et al. [148] also synthesized multi-heteroatom-doped defect-enriched CNTs (MH-DCNTs) catalyst by using aggressive oxidation of CNTs and followed high-temperature heteroatom doping. By unzipping and length shortening of CNTs, many lattice defects with carbon vacancies are in the presence of MH-DCNTs. Electrochemical analysis has shown that both ORR kinetic current density and onset potential of MH-DCNT were enhanced with the combination of lattice defect and heteroatom dopants. When employed MH-DCNTs as cathode catalyst in an anion exchange membrane fuel cell, the peak power density of the assembled device could be up to 250 mW cm^−2^, which was almost 70% of the performance of the conventional Pt/C electrode.

Remarkably, the heteroatom doping and defect-inducing co-engineering strategy is also suitable to boost the electrocatalytic activity for OER and HER, enabling as advanced bi- or trifunctional nanocarbon electrocatalysts. For example, Jiang et al. [33] developed defect-rich and ultrathin N-doped carbon nanosheets by a spontaneous gas-foaming method that exhibited an excellent trifunctional electrocatalytic activities for the ORR, OER, and HER. By means of DFT computations, it revealed that the most electrocatalytic active sites for the ORR, OER, and HER are the carbon atoms located at the armchair edge connected with the graphitic N dopants. Dai et al. [149] prepared 2D N-, S-codoped graphitic nanosheets with unique hierarchical porous structure consisting of stereoscopic holes that ensure it with high surface area and abundant interfacial/surface defective active sites for electrochemical reactions. Electrochemical measurements were demonstrated to be an effective trifunctional ORR, OER, and HER electrocatalyst with excellent activity and stability, which outperform the counterparts without or less opened holes and defective sites. Similarly, Hou et al. [143] reported N-, B-codoped graphitic carbon nanocage (NB-CN) with defect-rich property, which is a promising trifunctional electrocatalyst for ORR, OER, and HER. Furthermore, the P/N [73, 82, 125], O/N [114]-codoped, P/S/N [150, 151]-tridoped porous nanocarbons with abundant edges or defects have been widely employed as the bi- or trifunctional nanocarbon electrocatalysts for ORR, OER, and HER. These results demonstrated that the co-engineering of heteroatom doping and defect inducing is a general strategy for the enhancement of the electrocatalytic activities for nanocarbon.

### Correlation Among Defects, Dopants, and ORR Activities

**Fig. 10:**
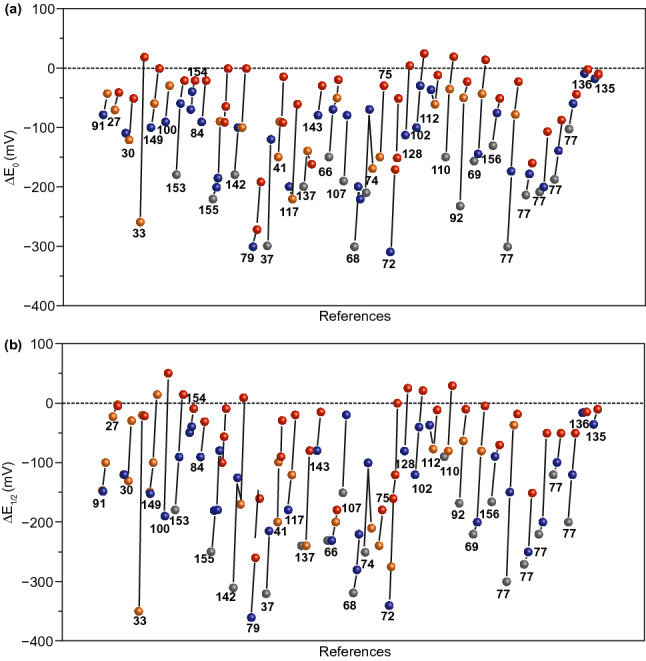
Comparison results of the ORR activities with their related commercial Pt/C catalyst: **a** Δ***E***_0_, **b** Δ***E***_1/2_; the gray, orange, blue, and red balls represent the pristine, defective, heteroatom-doped, and defect-/doping-co-engineered non-metal nanocarbon ORR electrocatalysts, respectively

**Fig. 11:**
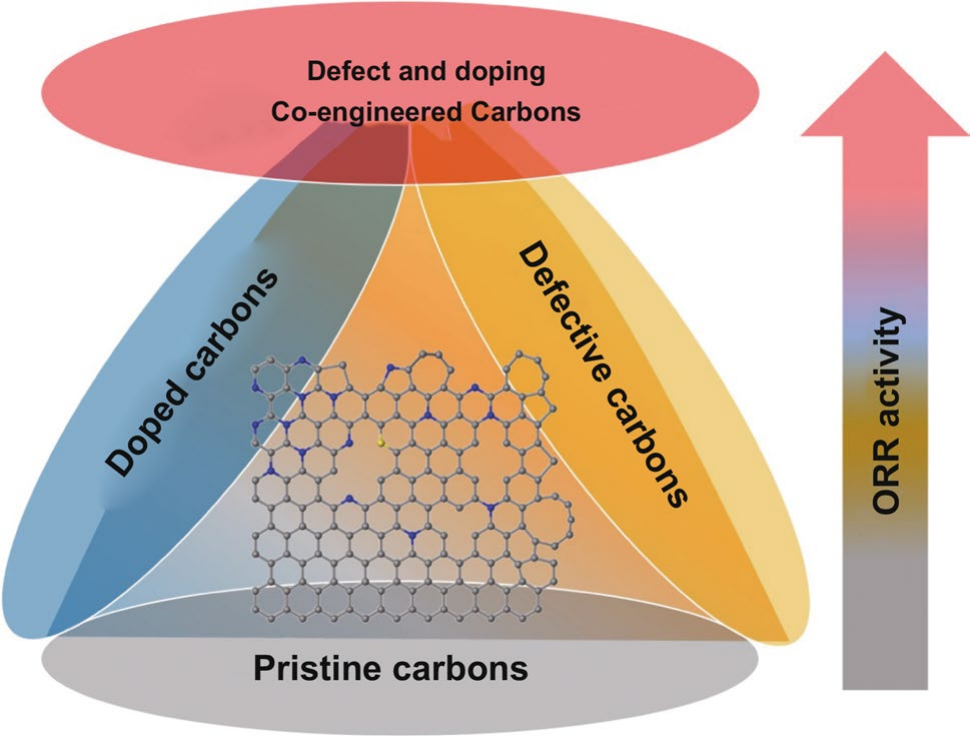
Trend of ORR activity of various functionalized non-metal carbon nanomaterials

As described in the previous sections, the electrocatalytic ORR activities of the carbon nanomaterials are highly associated with both defects and heteroatom dopants. Herein, in order to investigate the general rule of the various modified nanocarbon ORR electrocatalysts, we made an ORR activities comparison based on the differences between their ORR onset/half-wave potentials (Δ*E*_0_ and Δ*E*_1/2_, the two key parameters for ORR) and the related performance of commercial Pt/C catalysts, as roughly derived from their ORR polarization curve in the literature, since Pt-based materials (in most cases of the commercial Pt/C catalyst, 20%) are considered as the current state-of-the-art ORR catalyst, which is usually employed as the benchmark and the comparison object toward ORR. As exhibited in Fig. 10a, b, the non-modified pristine carbon nanomaterials, such as graphite, graphite nanoplates, graphene, fullerene, and CNTs, have little or even no activity toward ORR. After heteroatom doping or defect introducing, their ORR activities were improved. Most heteroatom-doped carbon materials describe higher ORR activity than defect-induced ones, while only a few are opposite. So it is difficult to predict which strategy is more facility for enhancing the ORR activity for the nanocarbons. Remarkably and undeniably, when combined with heteroatom doping and defect inducing, they usually reveal the best ORR performance, though there are a few examples to the contrary. Thus, the overall trend of electrocatalytic ORR activity of various functionalized carbon nanomaterials is approximately in accordance with Fig. 11, in which the defect- and doping-co-engineered nanocarbons reveal the highest ORR activity, the individual defect-enriched or heteroatom-doped carbons take the second place, and pristine carbons are the lowest among them. Table 1 presents the part of recent progress on advanced carbon-based metal-free ORR electrocatalysts by co-engineering of various defects and heteroatom dopants. Interestingly, their ORR performance can be comparable to or even outperformed than that of the state-of-the-art Pt/C catalyst, further confirming the synergistic promotion effect between defects and dopants.

**Table 1:** Typical defect- and heteroatom doping-co-engineered non-metal nanocarbon electrocatalysts and their key ORR parameters (onset and half-wave potentials, ***E***_0_ and ***E***_1/2_) in 0.1 M KOH solution

Catalysts	*E*_0_ (V vs. RHE)	*E*_1/2_ (V vs. RHE)	References
N-doped ultrathin carbon nanosheets	0.95	0.82	[33]
N, S codoped graphitic sheets with stereoscopic holes	1.01	0.87	[149]
N-doped graphene nanoribbons with interconnected 3D architecture	0.92	0.84	[154]
N-doped holey graphene	0.91	0.83	[41]
N,B-codoped graphitic carbon nanocage	0.92	0.83	[143]
N-doped porous carbon	0.92	0.82	[84]
Hierarchically porous N-doped carbon	0.94	0.87	[72]
N-doped nanocarbon with abundant oxygen-induced vacancies	0.93	0.86	[157]
Defect-enriched and S/N dual-heteroatom-doped graphene-like nanocarbons	0.95	0.85	[128]
Graphene-like nanobubble and nanosheet hybrid	0.97	0.84	[102]
3D few-layer N-doped porous carbon nanosheets	− 0.03 (vs. SCE)	− 0.17 (vs. SCE)	[110]
Pyridinic-N-dominated and defect-enriched graphene-like nanocarbon	0.99	0.87	[77]
Pentagon defect-rich N-doped carbon nanomaterial	0.91	0.83	[92]
Biomass-derived porous N-doped graphene	− 0.01 (vs. SCE)	− 0.20 (vs. SCE)	[136]

*RHE* reversible hydrogen electrode, *SCE* saturated calomel electrode

As known, the sites with the positive charge and/or higher spin density tend to chemically absorb oxygen molecular and the related intermediates, and subsequent electron transfer for O–O bond breaking and the electrocatalysis [39, 51, 90, 127, 152, 153, 155]. Accordingly, both of heteroatom dopants and defects can effectively modulate the charge and spin distribution of the carbon matrix at the level of electron structure, leading to the origin of electrochemical activities. Different configurations of heteroatom dopants and various defects contribute to the discriminative electron structure formation, thereby distinct electrocatalytic activities. A greatly promising strategy is proposed for the optimal design of carbon-based ORR catalysts via favorable doping at the specific defective sites of nanocarbon. Moreover, the heteroatom doping such as N-doping can facilitate the electroconductivity of the carbon materials [16, 20, 22]. The exposed edges and defective sites can increase the active density during the electrochemical reactions [25, 26, 52, 90]. Thus, to obtain carbon-based metal-free ORR catalysts with high active density and outstanding performance, the defects and heteroatom dopants should be simultaneously considered.

## Conclusion and Outlooks

The exploration of advanced metal-free carbon-based ORR catalysts is of great importance for the development of future clean energy, such as fuel cells and metal–air batteries. Significant progress of the ORR performance on the heteroatoms (N, P, S, B, etc.)-doped and/or various defects-induced carbon nanomaterials has been achieved in recent years. The incorporation of heteroatom dopants and construction of various defective sites are demonstrated to the two most promising approaches to the enhancement of the electrocatalytic ORR performance for metal-free nanocarbons. Both of them can efficiently reconstruct the *π* conjugation of carbon atoms, and induce the charge/spin redistribution or structural distortion of the surrounding C atoms to create electrocatalytic active sites, thereafter boosting the ORR kinetics. Remarkably, the combination of heteroatom doping and defect introducing can synergistically lower the reaction free energy and overpotential, endowing those nanocarbons with outperformed ORR activity (Fig. 10 and Table 1).

Be that as it may, it is still unclear which combinations or forms in carbon nanomaterials are the best for the electrocatalytic ORR. Based on the experimental results and theoretical predictions, the ideal active sites are the most likely to be adjacent to the combination of heteroatom dopants and the specific defects, such as N-doped graphene mesh with topological pentagon–heptagon pair and pentagon defects [25, 30], pyridinic-N adjacent to edge pentagon defect [69, 77], graphitic N and thiophene S co-modified edge pentagon defect [122]. Therefore, the targeted heteroatom-doped species along with dominated specific type of defects such as edge pentagon is expected to be the most effective for the construction of outperformed nanocarbon ORR electrocatalyst. However, there could be thousands and tens of thousands of combinations since the distribution and type of defective sites and heteroatom dopants in carbon nanomaterials are random and diverse. Fortunately, as the great progress and rapid development of computer technology, the use of high-throughput screening and machine learning technology in searching for the best active site for ORR may be the most efficient way. Besides, other significant challenges still exist, which are worthy of our further attention to developing the highly efficient nanocarbon ORR electrocatalysts.

1. Synergistic promotion mechanism of heteroatom doping and manufacturing defects. As described above, in most cases, the carbon nanomaterials with the combined heteroatom dopants and various defects could generate the best ORR activities. But the mechanism of how do they interact with each other and co-promote the electrocatalytic ORR activity has not been understood. Normally, the theoretical simulations are employed to reveal the correlation with the electronic structure, adsorption properties, and electrocatalytic activity. However, there is a big difference between the practical application and theoretical modeling simulation. Combined with more powerful and effective experimental characterizations, even in situ or operando techniques are hence necessary to observe the real process of electrocatalysis, which will be very helpful to further understand the mechanism of the electrocatalytic reaction.
2. How to precisely synthesize heteroatom dopants and the dominated specific defects in carbon nanomaterials remains a big challenge. Most of the reported carbon nanomaterials contain various defects and many types of dopant; it is therefore difficult to unveil the practical active sites and ORR mechanism. Creating the one or dominated specific type of defect with the targeted heteroatom-doped species can help us study the relationship between the dopants/defects and the electrochemical activity more systematically and clearly. Obviously, conventional pyrolysis method cannot meet the requirements, exploring newly efficient and controllable synthetic methods that need to be urgently developed.
3. Advanced characterization of specific defects. For heteroatom dopants in carbon materials, the content and types can be characterized by various spectroscopy analyses, such as XPS, ultraviolet photoelectron spectroscopy, X-ray absorption spectroscopy, and electron energy loss spectroscopy. For defects, Raman and positron annihilation techniques can only and roughly characterize the density and the types. The advanced TEM technologies could directly observe the defects, but it is only a part of various defects or a representative in carbon materials. There is still difficult to precisely quantify the concentration or ratio of a particular defect in carbon-based materials.
4. Applications. The electrocatalytic ORR activity of metal-free carbon-based nanomaterials in alkaline conditions is usually higher than that in acidic conditions, which include the quite different absorption intermediates and rate-determining steps in acidic and alkaline media. The current carbon-based ORR electrocatalysts are hence normally used in alkaline solutions, but not in acidic electrolytes. Since most of the practical fuel cells are more promising and competitive in acidic conditions (e.g., high efficiency and stability, non-effect of carbon dioxide), exploring and synthesizing more effective acidic carbon-based ORR electrocatalysts is of great significance.

All in all, carbon-based materials due to the multiple advantages of low cost, high efficiency, good stability, and long lifetime will undoubtedly have a promising application in the ORR field. Along with the present achievements and more advanced characterization methods combined with in situ techniques, the role of defects and heteroatom dopants, as well as their synergistic effect in the electrocatalysis field, will be able to further understand at a deeper level. In addition, we do hope that these achievements can also be extended to other energy conversation and storage technologies and electrochemical process.
